# First episode of acute mania with psychotic symptoms in a 70-year-old patient: Organic brain syndrome or not?

**DOI:** 10.1192/j.eurpsy.2026.11069

**Published:** 2026-07-31

**Authors:** N. Zigic, A. Jusufović, A. Alibegović, J. Dugić, A. Tufekčić, N. Salkovic, B. Hasanović, L. Alić, M. Mirkovic Hajdukov, N. Džekić, H. Porobić-Jahić, R. Jahić, N. Aljukić, E. Bećirović

**Affiliations:** 1 Department of Psychiatry, University Clinical Center; 2 School of Medicine, University of Tuzla, Tuzla; 3 Health Center, Žepče; 4 Health Center, Gračanica; 5 Public Health Department; 6 Department of Surgery, University Clinical Center; 7 Department of Psychology, Faculty of Humanities and Social Sciences; 8 Department of Infectious Diseases, University Clinical Center, Tuzla, Bosnia and Herzegovina

## Abstract

**Introduction:**

First onset of mania in older adults is unusual, and not often mentioned in literature. Studies suggest that new-onset mania in older adults is most commonly secondary due to pharmacological, metabolic or neurologic causes.

**Objectives:**

Present the case report of a 70-year-old patient with an acute manic episode without previous health issues.

**Methods:**

Psychiatric interview, laboratory tests, neuroradiological, microbiological and immunological examinations.

**Results:**

A 70-year-old patient was admitted to a psychiatric hospital for treatment due to altered behavior accompanied by agitation, thought process disorder, presence of paranoid delusions and increased sexual urges. The family provides information that the symptoms have lasted for about a year, but were intense 10 days before admission. In the first days of hospitalization, in addition to psychotic symptoms, strong affective irritability to the level of affective incontinence was noted, which led us to suspect the development of brain damage in the frontotemporal lobe. Information was obtained from family members that the patient had never previously had mental difficulties or been treated by a psychiatrist or other specialists. So far, he has been mostly healthy. Diagnostic tests were performed. Brain CT is completely normal. Laboratory findings show significantly elevated eosinophil values. The patient told us that approximately 8 months ago he ate pork that was not sufficiently heat-treated. An infectious disease specialist was consulted. Microbiological tests for trichinosis, leptospirosis, borreliosis, brucellosis and Q-fever were performed, as well as a series of immunological tests, of which IgE was elevated up to 160. All other tests were negative, except for elevated IgM antibodies to leptospirosis. Further infectious treatment is ongoing. The patient was treated with a mood stabilizer and antipsychotic in lower doses, which resulted in the cessation of psychomotor restlessness, reduction in delusional content and stabilization of affect. A double diagnosis of mania with psychotic symptoms and leptospirosis was made.

**Conclusions:**

Although rare, first episodes of mania with or without psychotic symptoms also occur in the elderly population and are most often secondary, therefore a detailed diagnostic work-up and careful titration of psychopharmacotherapy is required.

**Disclosure of Interest:**

None Declared

